# Prospective Evaluation of Mango Fruit Intake on Facial Wrinkles and Erythema in Postmenopausal Women: A Randomized Clinical Pilot Study

**DOI:** 10.3390/nu12113381

**Published:** 2020-11-04

**Authors:** Vivien W. Fam, Roberta R. Holt, Carl L. Keen, Raja K. Sivamani, Robert M. Hackman

**Affiliations:** 1 Department of Nutrition, UC Davis, Davis, CA 95616, USA; wfam@ucdavis.edu (V.W.F.); rrholt@ucdavis.edu (R.R.H.); clkeen@ucdavis.edu (C.L.K.); 2 Department of Internal Medicine, UC Davis, Sacramento, CA 95817, USA; 3 Department of Dermatology, UC Davis, Sacramento, CA 95816, USA; raja.sivamani.md@gmail.com; 4 Department of Biological Sciences, California State University, Sacramento, CA 95819, USA; 5 Pacific Skin Institute, Sacramento, CA 95815, USA; 6 Zen Dermatology, Sacramento, CA 95819, USA

**Keywords:** mango, skin, wrinkles, fruit, carotenoids, polyphenols, postmenopausal, dermatology, photoprotection

## Abstract

Mangos are rich in β-carotene and other carotenoids, along with several phenolic acids that may provide oxidant defense and photoprotection to the skin. The objectives of this study are to investigate the effects of Ataulfo mango intake on the development of facial wrinkles and erythema. A randomized two-group parallel-arm study was conducted to assess 16 weeks of either 85 g or 250 g of mango intake in healthy postmenopausal women with Fitzpatrick skin type II or III. Facial photographs were captured at weeks 0, 8, and 16, and wrinkles at the lateral canthi and erythema at the cheeks were quantified. Skin carotenoid values were measured with reflection spectroscopy. Deep wrinkle severity decreased significantly in the 85 g group after 8 (*p* = 0.007) and 16 (*p* = 0.03) weeks compared to baseline measures. In contrast, those in the 250 g group showed an increase after 16 weeks in average wrinkle severity (*p* = 0.049), average wrinkle length (*p* = 0.007), fine wrinkle severity (*p* = 0.02), and emerging wrinkle severity (*p* = 0.02). Erythema in the cheeks increased with 85 g of mango intake (*p* = 0.04). The intake of 85 g of mangos reduced wrinkles in fair-skinned postmenopausal women, while an intake of 250 g showed the opposite effect. Further studies feeding 85 g of mangos are warranted.

## 1. Introduction

Skin aging is generally classified as intrinsic and extrinsic. Intrinsic aging includes genetic factors that influence pigmentation of the skin, skin composition and thickness, and hormonal composition [1]. Extrinsic aging leads to premature aging of the skin and includes lifestyle and environmental factors such as smoking, temperature and humidity, and ultraviolet (UV) radiation [1]. Photoaging involves changes in the skin induced by repeated exposure to UV radiation and is most often seen as the primary form of extrinsic aging [2]. Wrinkles are a common result of these factors [3].

Numerous dietary factors can modulate skin health. For example, consumption of carotenoid-rich kale extracts in humans reduced radical formation, prevented collagen I degradation, and improved the extracellular matrix [4,5], while supplementation with both carotenoids and vitamin C has been reported to decrease wrinkles at the lateral canthi and improve skin hydration [6,7]. Consumption of lycopene-rich tomato nutrient complex and lutein in humans has also been shown to provide protection against damage from UV radiation at the molecular level [8]. Beta-carotene and other carotenoids may provide photoprotection through direct chemical reactions with UV-induced reactive oxygen species (ROS) and interfere with UV-induced gene expression [9]. In addition, β-carotene can be converted into Vitamin A in the liver. The role of Vitamin A and ascorbic acid on skin health is widely recognized and has been recently reviewed elsewhere [10]. Ascorbic acid is present in high concentration in the skin and functions both as a powerful reducing agent to combat the effects of ROS and UV radiation and is essential in the synthesis of collagen [10]. Vitamin A helps to regulate cellular differentiation by increasing epidermal proliferation to counteract photoaging [10].

Mangos may be particularly suited to provide compounds that benefit the skin, particularly Ataulfo mangos, which have a robust carotenoid profile (mainly β-carotene), along with phenolic acids and ascorbic acid, which are generally higher compared to other mango cultivars typically found in the US [11,12]. Gallic acid, chlorogenic acid, protocatechuic acid, and vanillic acid are the major phenolics identified in Ataulfo mangos [13]. Mice fed gallic acid demonstrated photoprotection by a reduction in the degradation of collagen [14], and the consumption of chlorogenic acid from coffee was correlated with lower UV pigmented spots in middle-aged Japanese women [15]. Mangos also contain mangiferin; a xanthone reported to possess anti-inflammatory properties and provide protection against UV radiation [16]. In a study with human cadaver skin, elastase and collagenase activity were inactivated by mangiferin in a dose-dependent manner [17].

While a mango extract fed to mice inhibited UVB-induced wrinkle formation through inhibition of epidermal thickening and increasing collagen bundles, the effects of mango consumption on human skin remain unknown [18]. We therefore assessed the effects of Ataulfo mango intake at 85 g (0.5 cup) or 250 g (1.5 cups) for 16 weeks on the development of wrinkles and erythema, and changes in skin carotenoids, in postmenopausal women.

## 2. Materials and Methods

### 2.1. Participants

Healthy postmenopausal women aged 50 to 70 were recruited from the greater Sacramento area. Inclusion criteria were Fitzpatrick skin type I, II, or III, and a body mass index (BMI) between 18.5 and 35 kg/m^2^. Exclusion criteria included allergy to mangos, self-reported malabsorption, daily intake of more than two cups of fruits and three cups of vegetables, fruit juice consumption of more than one cup per day, use of statins or anti-inflammatory drugs, medical or cosmetic procedures to the face within the past six months, current or recent (less than one year) cigarette smoking, and the use of antioxidant supplements. This intervention was conducted between August 2018 and June 2019 and was registered at ClinicalTrials.gov (NCT03590756), with the protocol approved by the Institutional Review Board of the University of California, Davis (IRB #1185928). All participants gave their informed consent for inclusion before they participated in the study.

### 2.2. Study Design

Eligible participants were randomized by block design into an open-label, two-arm parallel clinical trial consuming either 85 g or 250 g of Ataulfo mango, four times per week for 16 weeks. A no-mango control group was not utilized as no human studies investigating the effects of fresh-frozen mango intake on skin exist, and thus relevant reference data was unknown. The two amounts of mango used in the present study were based on the 2015–2020 Dietary Guidelines for Americans [19]. The 250 g (1.5 cups) amount was close to the recommendation of two cups of fruit per day, while still allowing participants to consume other fruits during the 16-week intervention. The 85 g (0.5 cup) comparison amount was selected to provide sufficient differentiation from the 250 g portion size. The duration of the intervention was established to allow sufficient time for a new dermal and epidermal layer of skin to develop. Study assessments were conducted in the morning after a 12-h fast at weeks 0, 8, and 16 in the Ragle Human Nutrition Center at the University of California, Davis, CA, USA.

### 2.3. Ataulfo Mangos

Fully ripe, fresh Ataulfo mangos were washed, peeled, cut, portioned, and immediately frozen and stored at −20 °C. Participants were given frozen mangos prepackaged into 85 g (0.5 cup) or 250 g (1.5 cups) daily servings and instructed to store them frozen and consume one serving, four times per week. Women could decide how to consume the mangos, as long as the fruit was not heated, since cooking could lead to losses in ascorbic acid and other vitamins [20].

### 2.4. Wrinkles and Erythema

**Figure 1:**
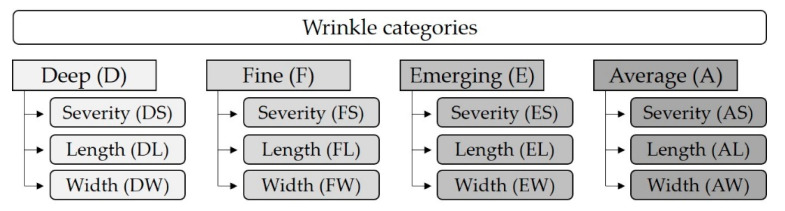
Classification of wrinkles. Deep wrinkles are high contrast, fine wrinkles are medium contrast, and emerging wrinkles are low contrast lines. Average wrinkles are the mean of all three categories. Severity is calculated as contrast multiplied by length.

High-resolution facial photographs were obtained in a dark room without skincare products or makeup (Mini Research 3D Clarity System, BrighTex Bio-Photonics, LLC, San Jose, CA, USA). Perspectives of the front, left, and right facial profiles were taken, rasterized, and classified based on the pixel image contrast. Lines with a minimum length of 2.6 mm with high contrast were termed deep (D), while those with medium contrast were grouped as fine (F) and with low contrast as emerging (E) (Figure 1). For each of these classifications, the wrinkle length (L) and width (W) were determined, along with a severity (S) score calculated as contrast multiplied by L. The average (A) values for L, W, and S were then calculated from the mean contrast of all pixels.

### 2.5. Skin Carotenoids

Skin carotenoids (SCs), units in mm wavelength, were measured in the right index finger after cleaning with alcohol by reflection spectroscopy (Veggie Meter^®^, Longevity Link Corporation, Salt Lake City, UT, USA). The device has been validated to correspond with plasma carotenoid levels and was calibrated before each use [21].

### 2.6. Blood Pressure and Lipids

Three blood pressure readings were obtained, five minutes apart after a fifteen-minute seated rest using an automated oscillometric unit and averaged to obtain systolic (SBP) and diastolic (DBP) values (Vital Spot, VSM 300, Welch Allyn, Skaneateles Falls, NY, USA). Plasma lipids were analyzed for cholesterol, low-density lipoprotein (LDL), non-high-density lipoprotein (HDL), and triglycerides at the UC Davis Department of Pathology and Laboratory Medicine.

### 2.7. Dietary Intake

A 24-h recall was conducted at each study visit using the validated Automated Self-Administered 24-h (ASA24) dietary assessment tool (https://epi.grants.cancer.gov/asa24). A compliance log was maintained, showing the date, time, and format of mango consumption.

### 2.8. Statistical Analysis

An a priori power analysis showed that there was greater than 80% power to detect a 10% difference in wrinkle severity between the 250 g and 85 g mango groups at Week 16, with recruitment of at least 15 subjects in each group with alpha set to *p* = 0.05. Statistical analyses were performed with JMP version 15 (SAS Institute Inc., Cary, NC, USA). *p* values of 0.05 or less were considered statistically significant. Each parameter was assessed for normality, or transformed (Log, Log10, or Johnson) to achieve normality before analyses. Data are presented as mean ± SD. Baseline participant characteristics were analyzed with the *t*-test or Wilcoxon signed-rank test as appropriate. All other outcome measures were analyzed using the Fit Model to perform Two Way ANCOVA, with treatment and time as factors, and BMI as a covariate. Post-hoc analyses were performed with Contrast tests. The Pearson and Spearman correlation coefficients were used to analyze the correlation between outcome measures that were normally and non-normally distributed, respectively.

## 3. Results

### 3.1. Baseline Characteristics

**Figure 2:**
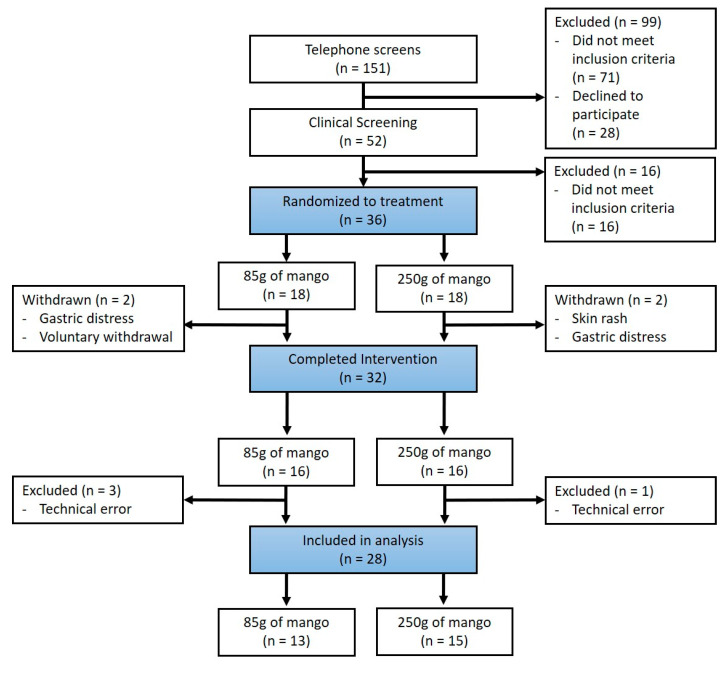
CONSORT flow diagram.

Thirty-six healthy postmenopausal women were enrolled. Thirty-two individuals completed the study (Figure 2). Analysis of skin carotenoids, blood pressure, and dietary records included all participants. For the wrinkle analysis, four sets of data were removed due to technical errors in image capture. Those that showed no deep wrinkles at baseline were removed from the analysis of both deep and the average wrinkle score (which used deep wrinkle values), resulting in data from eight and nine participants for the left lateral canthus, and ten and eight for the right lateral canthus for the 85 g and 250 g groups, respectively. Data for fine and emerging wrinkles were available from 13 to 15 individuals in the 85 g and 250 g groups, respectively. Analysis of plasma lipids and glucose included 21 participants (13 and eight in the 85 g and 250 g group, respectively).

At the start of the study, the two groups were similar in age, blood pressure, lipids, and fasting blood glucose (Table 1). Participants had either Fitzpatrick Skin Type II or III (*n* = 9 and 19, respectively). Baseline BMI values were significantly different, with the mean for those in the 85 g group were classified as overweight, while the average for the 250 g group was in the normal range. Left side measurements were significantly different between the two groups at baseline for fine (FL) and emerging wrinkle length (EL).

### 3.2. Dietary Intake

Both groups consumed approximately 1700 kcals at the baseline, with few significant differences in macronutrients (Table S1); the 85 g group had higher intakes of fiber, folate, and lutein plus zeaxanthin. After 16 weeks, the 85 g group reported increases in dietary potassium and cholesterol, while the 250 g group had a significant increase in total sugars, as well as potassium and folate.

### 3.3. Facial Wrinkles and Erythema

**Figure 3:**
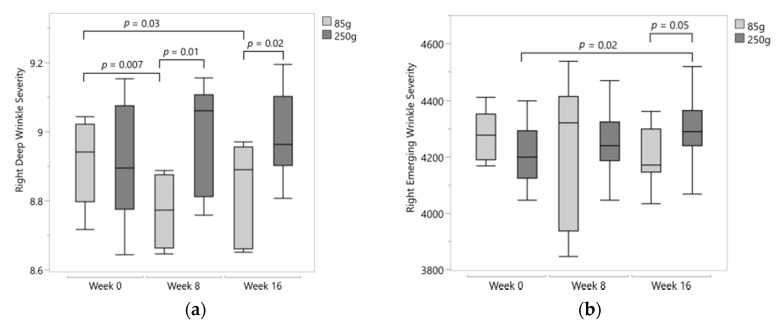
(**a**) Right deep wrinkle severity was significantly different between groups at week 8 (*p* = 0.01) and 16 (*p* = 0.02) and significantly decreased by week 8 (*p* = 0.007) and 16 (*p* = 0.03) in the 85 g group. Data transformed by natural log. (**b**) Right emerging wrinkle severity significantly increased (*p* = 0.02) by week 16 in the 250 g group. Data transformed by natural log.

At baseline, no differences were noted in right lateral canthi measures, while left FL and EL were significantly lower in the 250 g compared to the 85 g group (Table 1). In the 85 g group, right deep wrinkle severity decreased by 23% after eight weeks and 20% after 16 weeks (both of which were significant (*p* = 0.007 and *p* = 0.03, respectively; Figure 3a). A trend for reduced left deep wrinkle severity (DS) was noted in the 85 g group (severity scores at baseline: 7575 ± 1063 vs. 16 weeks: 5339 ± 3153, *p* = 0.10). In contrast, an increasing trend in right DS was observed in the 250 g group (severity scores at baseline: 7537 ± 1338 vs. 16 weeks: 8056 ± 982, *p* = 0.07). Comparison of the two groups showed a trend for lower left DS after 16 weeks in the 85 g group relative to the 250 g group (severity scores for 85 g: 5339 ± 3153 vs. 250 g: 8105 ± 1840, *p* = 0.08). For the right DS, the 85 g mango group was significantly lower compared to 250 g at both week eight (*p* = 0.01) and week 16 (*p* = 0.02; Figure 3a).

Deep wrinkle length (DL) in the 85 g group was reduced by 32.7% after 16 weeks compared to baseline (baseline: 17.61 ± 5.05 mm vs. 16 weeks: 11.85 ± 7.56 mm, *p* = 0.07) that was significantly lower compared to the 250 g group (85 g: 11.85 ± 7.56 mm vs. 250 g: 18.21 ± 5.91 mm, *p* = 0.02). A between-group trend for increased right deep wrinkle width (DW) (85 g: 1.44 ± 0.63 mm vs. 250 g: 1.74 ± 0.20 mm, *p* = 0.08) and right emerging wrinkle severity (ES) (severity scores at 85 g: 4203 ± 94 vs. 250 g: 4299 ± 108, *p* = 0.05) was observed after 16 weeks in the 250 g. These between-group differences in right ES may be attributed to a 2.9% increase from baseline in the 250 g group (*p* = 0.02; Figure 3b).

**Figure 4:**
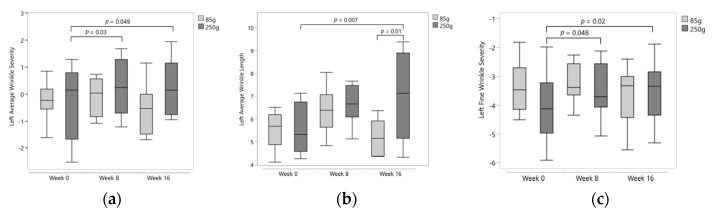
(**a**) Left average wrinkle severity significantly increased at week 8 (*p* = 0.03) and week 16 (*p* = 0.049) after 250 g of mango intake. Data transformed by Johnson transformation. (**b**) Left average wrinkle length significantly increased at week 16 (*p* = 0.007) after 250 g of mango intake and is significantly higher than the 85 g group (*p* = 0.01). (**c**) Left fine wrinkle severity significantly increased at week 8 (*p* = 0.048) and 16 (*p* = 0.02) after 250 g of mango intake. Data transformed by Johnson transformation.

A number of left side wrinkle measures significantly increased in the 250 g group, with no significant changes from baseline for the 85 g group. Compared to baseline, left average wrinkle severity (AS) significantly increased by week 8 (*p* = 0.03), which persisted to week 16 (*p* = 0.049; Figure 4a). A 25% increase from baseline in left average wrinkle length (AL) was observed (*p* = 0.007), which was also significantly higher than the value for the 85 g group at week 16 (*p* = 0.01; Figure 4b). Left fine wrinkle severity (FS) increased from baseline after 8 weeks (*p* = 0.048) and after 16 weeks (*p* = 0.02; Figure 4c).

For erythema measures, left cheek erythema was significantly increased after 16 weeks in the 85 g group (degree of intensity % at baseline: 21.2 ± 18.0 vs. 16 weeks: 27.2 ± 16.0, *p* = 0.04), while no changes were observed in the 250 g group.

### 3.4. Skin Carotenoids

At baseline, SCs were generally lower in the 85 g compared to the 250 g group (85 g: 363 ± 78 mm wavelength vs. 250 g: 432 ± 105 mm wavelength, *p* = 0.06). Mango intake did not result in a significant change within or between groups over time. While no significant changes in SCs were observed for those who were normal weight in either group, those who were overweight or obese showed a significant increase from baseline after eight (348 ± 59 mm wavelength, *p* = 0.01) and 16 (352 ± 76 mm wavelength, *p* = 0.03) weeks, regardless of group assignment. These findings may be due, in part, to the fact that participants who were overweight or obese had significantly lower SCs at baseline compared to those who were of normal weight (329 ± 77 mm wavelength vs. 452 ± 104 mm wavelength, respectively, *p* = 0.008).

### 3.5. Blood Pressure and Plasma Lipids

A significant decrease in SBP was observed in the 250 g group after eight and 16 weeks of intake, compared to baseline measures (baseline: 113 ± 2.28 mm Hg vs. 8 weeks: 108.5 ± 2.28 mm Hg, *p* = 0.02 and baseline vs. 16 weeks: 108.2 ± 2.28 mm Hg, *p* = 0.01). Similar declines were observed for DBP (baseline: 74.1 ± 1.41 mm Hg vs. 8 weeks: 71.2 ± 1.41 mm Hg, *p* = 0.01 and baseline vs. 16 weeks: 71.0 ± 1.41 mm Hg, *p* = 0.009), and mean arterial pressure (MAP) (baseline: 87.1 ± 1.65 mm Hg vs. 8 weeks: 83.6 ± 1.65 mm Hg, *p* = 0.01 and baseline vs. 16 weeks: 83.4 ± 1.65 mm Hg, *p* = 0.008). No significant interactive effects (treatment x time) for any of the blood pressure or plasma lipid measures were noted. No significant changes in blood pressure were observed for the 85 g group.

Serum cholesterol decreased significantly by 7.4% in the 250 g group at the end of the study (baseline: 216 ± 9.7 mg/dL vs. 16 weeks: 200 ± 9.7 mg/dL, *p* = 0.02), while trends were observed for decreasing LDL (baseline: 122 ± 7.6 mg/dL vs. 16 weeks: 108 ± 7.3 mg/dL, *p* = 0.06) and non-HDL (baseline: 138 ± 7.9 mg/dL vs. 16 weeks: 127 ± 7.9 mg/dL, *p* = 0.07). In the 85 g group, triglycerides were lower compared to baseline after 16 weeks (baseline: 70.6 ± 20.7 mg/dL vs. 16 weeks: 63.3 ± 36.1 mg/dL, *p* = 0.06).

No significant changes were observed in blood glucose with either 85 g or 250 g of mango intake after 16 weeks.

### 3.6. Correlations

Reported intake of dietary β-carotene was positively correlated with SCs (*r* = 0.22, *p* = 0.048) and with erythema in the right (*r* = 0.39, *p* = 0.0006) and combined cheek measurements (*r* = 0.39, *p* = 0.0009). In addition, dietary β-carotene intake was negatively correlated with blood pressure: SBP (*r* = −0.28, *p* = 0.01), DBP (*r* = −0.29, *p* = 0.008), and MAP (*r* = −0.29, *p* = 0.008). Skin carotenoids were positively correlated with erythema on the left (*r* = 0.24, *p* = 0.04) and right cheek (*r* = 0.40, *p* = 0.0005). Skin carotenoids were also negatively correlated with SBP (*r* = −0.37, *p* = 0.0006), DBP (*r* = −0.33, *p* = 0.002), and MAP (r = −0.34, *p* = 0.002).

## 4. Discussion

In this exploratory trial, we observed that 85 g (0.5 cup) of mango intake for two to four months reduced facial wrinkles while 250 g (1.5 cups) increased them. We report here a significant decrease in right DS and a trend in the reduction for left DS and DL in the 85 g group. In contrast, the 250 g group showed a significant increase in left AS, AL, FS, and right ES and trended towards an increase in right DS and DW. While further research is needed to explore the mechanisms behind these findings, the reduction in wrinkles with 85 g of mango intake may be due to the beneficial effects of carotenoids, flavonoids, and mangiferin, all of which, as part of a whole food complex, could lead to improvements in collagen bundles and a reduction in epidermal thickening as seen with the mouse study noted above [18]. The increase in wrinkles in the 250 g group was notable and unexpected. Since a significant increase in total sugar intake was noted in this group after eight and sixteen weeks, this increased sugar intake may have led to glycation of collagen fibers, thereby disrupting the collagen structure [22]. The effects of whole food intake on skin health in humans is a relatively new area of research. A recent study reported a significant reduction in facial wrinkles in postmenopausal women after regular intake of almonds [23]. Although dietary and skin carotenoids were positively correlated with erythema, a significant increase in erythema was only observed in the 85 g mango group. Therefore, it is unlikely that erythema was caused by the amount of β-carotene consumed from the fruit. Furthermore, β-carotene has been shown to decrease erythema in other studies [9,24].

Favorable changes in markers of cardiovascular risk were noted in the 250 g group. Serum cholesterol significantly decreased by 7.4%, while a trend for reduced LDL and non-HDL cholesterol was observed. This may possibly be attributed to plant sterols that are abundant in mangos (24.4 mg/100 g fruit), as these compounds have been associated with a reduction in LDL cholesterol [25,26,27]. The 250 g of mango intake may also have improved cholesterol levels by providing a significant source of both soluble (28.2 g/100 g dry matter) and insoluble (41.5 g/100 g dry matter) fiber [28,29]. Our data is consistent with the observation of reduced total and LDL cholesterol levels after Ataulfo mango pulp intake in a rat model [30].

We also observed a 4% reduction in blood pressure with higher levels of mango intake. The inverse correlations observed between dietary and skin carotenoids with blood pressure are consistent with findings from other studies that report negative correlations between β-carotene and SBP, as well as with cardiovascular mortality [31,32]. Taken together, these results suggest a potential role of mango intake on cardiometabolic health but require confirmation through future trials powered explicitly for these outcomes.

### Limitations

Two levels of mango intake were used in this exploratory study to determine whether either amount would produce a change in facial wrinkles. Thus, a control group consuming no mangos was not employed. However, a strength of the study is that the design allowed for the assessment of an amount-based response and allowed for better control of any placebo effect that could be present as women in both groups received mangos. Finally, the study was limited to healthy postmenopausal women with Fitzpatrick skin type II and III; therefore, the findings reported here may not be generalizable to other groups.

## 5. Conclusions

Results from this pilot study support the concept that regular intake of modest amounts of mangos may improve facial wrinkles. The apparent beneficial effects of mangos on skin health may be lost if the intake of mangos is particularly high. The effects of whole food intake on skin health are limited but promising. Further prospective studies are warranted.

## Figures and Tables

**Table 1:** Baseline characteristics of participants.

	85 g Group (*n* = 13)	250 g Group (*n* = 15)	*p*
	Mean ± SD	Mean ± SD	
Age (years)	61 ± 5.1	60 ± 5.3	0.58
BMI (kg/m^2^)	26.4 ± 4.0	22.9 ± 2.6	0.01 *
WC (cm)	89 ± 12.0	83 ± 9.4	0.13
SBP (mmHg)	115 ± 10.7	113 ± 6.9	0.96 ^†^
DBP (mmHg)	75 ± 6.5	74 ± 4.0	0.61
HR (BPM)	63 ± 6.2	61 ± 7.2	0.53
RS Carotenoid (nm wavelength)	363 ± 78	432 ± 105	0.06
Glucose (mg/dL)	97.5 ± 6.5	95.1 ± 7.5	0.46 ^‡^
Cholesterol (mg/dL)	220 ± 31.9	225 ± 44.2	0.83 ^‡^
LDL (mg/dL)	138 ± 25.6	127 ± 27.7	0.42 ^‡^
Non-HDL (mg/dL)	152 ± 24.7	143 ± 27.1	0.49 ^‡^
Triglycerides (mg/dL)	71 ± 21.6	83 ± 69.3	0.16 ^‡,†^
**Left lateral canthus**			
Deep Wrinkle Severity	7575 ± 1063.4	8037 ± 1403.2	0.46 ^Ŧ^
Deep Wrinkle Length (mm)	17.61 ± 5.05	17.68 ± 3.77	0.98 ^Ŧ^
Deep Wrinkle Width (mm)	1.63 ± 0.25	1.84 ± 0.24	0.09 ^Ŧ^
Fine Wrinkle Severity	5866 ± 680.4	5148 ± 1510.4	0.08 ^†^
Fine Wrinkle Length (mm)	5.49 ± 1.05	4.01 ± 1.40	0.004 *
Fine Wrinkle Width (mm)	1.46 ± 0.18	1.29 ± 0.40	0.24
Emerging Wrinkle Severity	4279 ± 123.8	4249 ± 110.2	0.51
Emerging Wrinkle Length (mm)	5.06 ± 2.92	3.64 ± 0.61	0.02 *^,†^
Emerging Wrinkle Width (mm)	1.20 ± 0.13	1.21 ± 0.13	0.90
Average Wrinkle Severity	5166 ± 379.1	5329 ± 639.3	0.54 ^Ŧ^
Average Wrinkle Length (mm)	5.71 ± 1.18	5.57 ± 1.08	0.80 ^Ŧ^
Average Wrinkle Width (mm)	1.28 ± 0.09	1.35 ± 0.18	0.47 ^Ŧ,†^
**Right lateral canthus**			
Deep Wrinkle Severity	7487 ± 922.1	7537 ± 1338.0	0.93 ^Ŧ^
Deep Wrinkle Length (mm)	16.62 ± 5.53	17.41 ± 6.27	0.78 ^Ŧ^
Deep Wrinkle Width (mm)	1.70 ± 0.20	1.71 ± 0.27	0.93 ^Ŧ^
Fine Wrinkle Severity	5730 ± 330.4	5778 ± 485.1	0.76
Fine Wrinkle Length (mm)	5.75 ± 3.07	4.91 ± 0.85	0.85 ^†^
Fine Wrinkle Width (mm)	1.52 ± 0.23	1.38 ± 0.15	0.08
Emerging Wrinkle Severity	4240 ± 160.6	4178 ± 186.9	0.18 ^†^
Emerging Wrinkle Length (mm)	4.00 ± 0.61	3.62 ± 0.52	0.09
Emerging Wrinkle Width (mm)	1.22 ± 0.12	1.26 ± 0.16	0.47
Average Wrinkle Severity	5169 ± 256.9	5336 ± 541.7	0.40 ^Ŧ^
Average Wrinkle Length (mm)	6.10 ± 1.84	6.12 ± 1.44	0.82 ^Ŧ,†^
Average Wrinkle Width (mm)	1.36 ± 0.13	1.38 ± 0.16	0.81 ^Ŧ^
**Erythema**			
Left Cheek	14.73 ± 6.90	16.18 ± 4.96	0.55
Right Cheek	18.02 ± 13.08	14.00 ± 8.85	0.43 ^†^

* Significantly different between 85 g and 250 g groups (*p* < 0.05). Statistical analysis by t test or ^†^ Wilcoxon’s signed-rank test. ^Ŧ^ Average and deep wrinkles measurements had an *n* of 8 and 9 in the left, and an *n* of 10 and 8 in the right, in the 85 g and 250 g group, respectively. ^‡^ Glucose measurement had an *n* of 12 and 9 participants for 85 g and 250 g group, respectively. BMI = Body Mass Index; WC = Waist Circumference; SBP = Systolic Blood Pressure; DBP = Diastolic Blood Pressure; HR = Heart Rate; RS = Reflection Spectroscopy; LDL = Low-density Lipoprotein; HDL = High-density Lipoprotein.

## Supplementary Materials

The following are available online at https://www.mdpi.com/2072-6643/12/11/3381/s1, Table S1: 24-h reported dietary intake.
