# Supplementary material for: Prospective Evaluation of Mango Fruit Intake on Facial Wrinkles and Erythema in Postmenopausal Women: A Randomized Clinical Pilot Study
Source: Nutrients. 2020 Nov 4;12(11):3381. doi: 10.3390/nu12113381 (PMC7694217; doi:10.3390/nu12113381)
Supplement: Supplementary file 1 [file nutrients-12-03381-s001.pdf]

Supplementary

Table S1. 24-hour reported dietary intake.

	85g group (n = 16)					250g group (n = 16)					Between groups		
	Week 0	Week 8	Week 16	*p* Week 8 vs 0	*p* Week 16 vs 0	Week 0	Week 8	Week 16	*p* Week 8 vs 0	*p* Week 16 vs 0	*p* Week 0	*p* Week 8	*p* Week 16
	Mean ± SD	Mean ± SD	Mean ± SD			Mean ± SD	Mean ± SD	Mean ± SD					
Energy (kcal)	1698 ± 555	1865 ± 575	1904 ± 801	0.21	0.37	1713 ± 440	2037 ± 549	1820 ± 827	0.12	0.75	0.76	0.93	0.21
Protein (g)	70 ± 37	83 ± 30	79 ± 30	0.09	0.29	74 ± 42	84 ± 33	69 ± 39	0.3	0.16	0.71	0.83	0.08
Total Fat (g)	70 ± 35	75 ± 35	90 ± 51	0.48	0.26	76 ± 25	95 ± 25	77 ± 50	0.21	0.17	0.69	0.37	0.07
Carbohydrates (g)	201 ± 45	208 ± 85	194 ± 81	0.81	0.58	180 ± 56	214 ± 71	220 ± 89	0.09	0.06	0.06	0.39	0.86
Sugars, total (g)	90 ± 30	111 ± 56	74 ± 38	0.22	0.13	78 ± 31	97 ± 31	107 ± 51	0.04*	0.01*	0.06	0.25	0.15
Fiber, total dietary (g)	26 ± 18	20 ± 7	22 ± 8	0.68	0.79	18 ± 6	26 ± 11	24 ± 11	0.03	0.13	0.02*	0.87	0.41
Potassium (mg)	2667 ± 770	3183 ± 1182	2718 ± 857	0.01*	0.99	2441 ± 846	3233 ± 746	3074 ± 1267	0.008*	0.049*	0.17	0.2	0.8
Vitamin C (mg)	99 ± 77	201 ± 276	171 ± 293	0.15	0.7	88 ± 67	140 ± 79	135 ± 97	0.02*	0.15	0.23	0.64	0.78
Folate, total (mcg)	401 ± 147	419 ± 243	466 ± 177	0.85	0.4	316 ± 118	467 ± 205	439 ± 171	0.005*	0.03*	0.02*	0.96	0.24
Vitamin A, RAE (mcg_RAE)	826 ± 438	996 ± 741	1046 ± 641	0.59	0.8	673 ± 362	1483 ± 1401	806 ± 540	0.04	0.69	0.29	0.63	0.36
Retinol (mcg)	315 ± 216	373 ± 206	397 ± 235	0.38	0.18	327 ± 122	386 ± 227	373 ± 265	0.59	0.48	0.51	0.74	0.2
Carotene, beta (mcg)	5614 ± 5746	6957 ± 8452	6935 ± 5539	0.61	0.89	3504 ± 3594	11154 ± 14647	4865 ± 6507	0.01	0.52	0.11	0.93	0.24
Carotene, alpha (mcg)	619 ± 740	935 ± 1149	1584 ± 2010	0.24	0.16	893 ± 1511	2661 ± 6638	496 ± 1143	0.24	0.38	0.56	0.53	0.12
Lutein + zeaxanthin (mcg)	5692 ± 8191	6934 ± 11437	5785 ± 6129	0.71	0.69	2289 ± 2418	5846 ± 12381	4000 ± 5678	0.61	0.74	0.03*	0.17	0.13
Vitamin E, alpha-tocopherol (mg)	10.8 ± 7	10.2 ± 4.5	11.2 ± 5.3	0.6	0.93	9.5 ± 3.8	17.4 ± 10.4	13.4 ± 10.1	0.008	0.27	0.23	0.44	0.88
Cholesterol (mg)	314 ± 283	330 ± 211	414 ± 276	0.51	0.04*	282 ± 218	264 ± 163	212 ± 193	0.82	0.046*	0.29	0.77	0.01*
Fatty acids, total saturated (g)	19 ± 12	22 ± 12	27 ± 22	0.38	0.29	25 ± 9	27 ± 9	23 ± 15	0.53	0.07	0.15	0.22	0.28
Fatty acids, total monounsaturated (g)	26 ± 14	27 ± 12	34 ± 18	0.45	0.25	26 ± 11	36 ± 11	29 ± 21	0.08	0.5	0.91	0.43	0.08
Fatty acids, total polyunsaturated (g)	20 ± 9	19.5 ± 10.7	23 ± 10	0.96	0.49	20 ± 8.5	26 ± 12	19 ± 13	0.34	0.17	0.68	0.59	0.02*

*Significant at the $p < 0.05$ level. Analyzed with Two Way ANCOVA and BMI as a covariate.
